# Clinical and Molecular Features of Thiazide-Induced Hyponatremia

**DOI:** 10.1007/s11906-018-0826-6

**Published:** 2018-04-10

**Authors:** Jodie Nadal, Sarath K. Channavajjhala, Wenjing Jia, Jenny Clayton, Ian P. Hall, Mark Glover

**Affiliations:** 1 Division of Respiratory Medicine, Queen’s Medical Centre, University of Nottingham, Nottingham, NG7 2UH UK; 2 Division of Endocrinology, Nottingham University Hospitals NHS Trust, Nottingham, UK; 3 NIHR Nottingham Biomedical Research Centre, Nottingham, UK

**Keywords:** Hyponatremia, Thiazide-induced hyponatremia, TIH, Hypertension, Thiazide, Diuretics

## Abstract

**Purpose of Review:**

Hypertension affects more than 30% of the world’s adult population and thiazide (and thiazide-like) diuretics are amongst the most widely used, effective, and least costly treatments available, with all-cause mortality benefits equivalent to angiotensin-converting enzyme inhibitors or calcium channel antagonists. A minority of patients develop thiazide-induced hyponatremia (TIH) and this is largely unpredictable at the point of thiazide prescription. In some cases, TIH can cause debilitating symptoms and require hospital admission. Although TIH affects only a minority of patients exposed to thiazides, the high prevalence of hypertension leads to TIH being the most common cause of drug-induced hyponatremia requiring hospital admission in the UK. This review examines current clinical and scientific understanding of TIH. Consideration is given to demographic associations, limitations of current electrolyte monitoring regimens, clinical presentation, the phenotype evident on routine clinical blood and urine tests as well as more extensive analyses of blood and urine in research settings, recent genetic associations with TIH, and thoughts on management of the condition.

**Recent Findings:**

Recent genetic and phenotyping analysis has suggested that prostaglandin E2 pathways in the collecting duct may have a role in the development of TIH in a subgroup of patients. Greater understanding of the molecular pathophysiology of TIH raises the prospect of pre-prescription TIH risk profiling and may offer novel insights into how TIH may be avoided, prevented and treated.

**Summary:**

The rising prevalence of hypertension and the widespread use of thiazides mean that further understanding of TIH will continue to be a pressing issue for patients, physicians, and scientists alike for the foreseeable future.

## Introduction to the Role of Thiazides in the Treatment of Hypertension

Hypertension is the most common modifiable cause of cardiovascular disease—the leading cause of mortality worldwide [1, 2]. At least 30% of the UK adult population have hypertension, and treatment consumes over a billion pounds per year in NHS drug costs alone [1, 3]. Whilst the treatment of hypertension is both evidence based and cost-effective [1], most patients require multiple antihypertensive agents, side effects are commonplace, and blood pressure control for many remains poor [3]. The current therapeutic limitations are perhaps not surprising since for most affected individuals we are still unable to define either the molecular mechanisms driving their hypertension or those leading to adverse effects from antihypertensive treatment.

The association of hypertension with salt is intriguing however; sodium chloride intake correlates with the prevalence of hypertension even in disparate populations [4], and sodium restriction lowers blood pressure, especially in those already hypertensive [5]. The physiological effects of salt on blood pressure are not a simple function of intake but reflect the balance of dietary salt ingestion and its excretion into the urine. Regulation of sodium reabsorption from tubular filtrate in the distal nephron has a marked effect on blood pressure and although the amiloride-sensitive epithelial sodium (Na) channel (ENaC) has classically dominated research interest; Na-Cl reabsorption via the thiazide-sensitive sodium chloride co-transporter, NCC, is at least as important [6].

Thiazide and thiazide-like diuretics share a common mechanism of action in the distal convoluted tubule of the nephron by binding to the luminal side of the thiazide-sensitive sodium chloride co-transporter, NCC. NCC is responsible for around 5–7% of renal sodium reabsorption [7]. Commonly used examples of this group of medicines include the thiazide diuretics bendroflumethiazide and hydrochlorothiazide, and the thiazide-like diuretics indapamide and chlortalidone. Thiazide diuretics are potent antihypertensive agents [1] and mimic the effects of loss of function NCC mutations seen in the hypotensive Mendelian syndrome of Gitelman [8]. Moreover, the heritable condition of hyperkalemic hypertension, Gordon syndrome (GS), results from increased sodium reabsorption via NCC and is effectively treated by low-dose thiazide diuretics and/or dietary sodium restriction [9].

Since the demonstration of their antihypertensive effect in 1958 [10], thiazides have been widely used in the management of hypertension. Their benefits on all-cause mortality are equal to those of angiotensin-converting enzyme (ACE) inhibitors and calcium channel antagonists [11, 12]. Thiazide and thiazide-like diuretics continue to be used first line in most countries in 2018 including the USA [13] and Europe [14]. Their recent demotion to step 3 in UK hypertension guidance has been controversial [1, 15].

Despite the clinical success of thiazides, they are not a panacea; they are often ineffective as monotherapy for essential hypertension [1] and cause significant side effects of which hyponatremia is amongst the most important, both clinically and scientifically as a paradigm of dysregulation of sodium (and water) reabsorption in the kidney [16]. Both the effectiveness of thiazide diuretics in essential hypertension and in GS and their limitation in the form of thiazide-induced hyponatremia (TIH) afford valuable opportunities to probe the molecular pathophysiology of salt reabsorption in the distal nephron and to inform the judicious use of thiazides in the treatment of hypertension.

In this review, we will concentrate on two principal sources of clinical data: a systematic review of all published TIH cases up to 2013 [17••] and a subsequent series of 109 patients admitted to hospital with severe TIH published in 2017 [18••]. The former has the advantage of being a comprehensive and systematic literature review which identified > 2800 patients with TIH, and the latter is a large contemporary case series in which genetic and extended phenotyping were undertaken, including assessment of TIH patients after recovery to normonatremia as an approximation of their likely baseline physiology.

## Clinical Features of TIH

A minority of patients exposed to thiazides develop a sufficiently reduced serum sodium concentration to be defined as having TIH. However, because hypertension is such a prevalent condition, TIH is the most common cause of drug-induced hyponatremia requiring admission to hospital [19•]. Classification of degrees of hyponatremia is inconsistent but serum sodium 130–134 mM is often termed mild, 125–130 mM moderate, and < 125 mM severe.

A systematic review and meta-analysis of all published cases of TIH to 2013 found that the mean age of patients with TIH was 75 (95% CI 73–77) years, 78% were women (95% CI 74–82), and their mean body mass index was 25 (95% CI 20–30) kg/m^2^ [17••]. A subsequent large case series of 109 patients hospitalized with severe TIH broadly agreed with this, with mean age 80 ± 9 years, 70% women, and BMI 25.7 ± 0.6 [18••]. Even when patients had recovered from TIH and were normonatremic, their weight remained lower than those of control patients who had not developed TIH, suggesting that reduced body mass may be associated with increased risk of development of TIH [18••]. Both systolic and diastolic blood pressure was lower in acute TIH compared to that in normonatremic controls and convalescent TIH cases. The biological significance of gender is unclear since the mean age of those with TIH puts them well beyond the menopause, perhaps implying a limited role for oestrogens.

Symptoms of TIH range from none or very mild to severe and life threatening (Table 1). The severity of TIH symptoms is likely to be related both to the rate of reduction in serum sodium and the absolute degree of hyponatremia; patients who develop a rapid reduction and very low absolute sodium concentration have the most severe symptoms; conversely, those who develop hyponatremia slowly and with modest reduction in absolute serum sodium concentration are more mildly affected.

**Table 1:** Meta-analysis of the symptoms reported at presentation in patients with thiazide-induced hyponatremia [17••]. Prevalence estimates from meta-analysis and confidence intervals are all expressed as proportions. *Prop*, proportion; *Pop* contributing population to the meta-analyses, number of studies/total number of patients with the studies; *CI*, confidence interval; *N*, number of single case reports reporting the variable listed

Symptoms	Prop	95% CI	*I*^2^ (%)	Pop	Summary of case report data	
				Studies/patients	*N*	%
Falls	0.53	0.17 to 0.88	88	4/241	2	4
Fatigue	0.46	0.21 to 0.72	92	8/333	18	38
Weakness	0.45	0.32 to 0.58	49	14/247	13	27
Confusion	0.44	0.32 to 0.56	85	21/699	16	33
Nausea	0.37	0.24 to 0.50	78	13/394	10	21
Neurological symptoms	0.36	0.20 to 0.54	13	7/26	10	21
Vomiting	0.35	0.25 to 0.46	71	12/538	9	19
Dizziness	0.31	0.15 to 0.51	92	8/488	4	8
Unconsciousness	0.30	0.15 to 0.48	75	11/181	13	27
Seizures	0.19	0.08 to 0.34	84	9/394	10	21

TIH has been reported with many types of thiazide and thiazide-like diuretics [17••, 18••] and it is unclear whether any particular thiazide medication is any more likely than the others to cause TIH. Dose dependency is also unclear although it is certainly possible to develop severe TIH at the lowest prescribable dose and frequency of individual thiazide or thiazide-like medicines.

Comorbidities and polypharmacy are important clinical and scientific considerations in TIH since these are factors which often affect the elderly population. The most frequent comorbidities in TIH identified by systematic review and meta-analysis were cardiovascular disease (49%, 95% CI 33 to 65%) and diabetes mellitus (27%, 95% CI 14 to 42%) although there was substantial heterogeneity between studies which was not explained by quality score, year of publication or age of patient [17••]. The 2017 case series of TIH patients broadly agreed with this having 17% of patients with treated diabetes mellitus, 19% with moderate renal impairment (eGFR 30–60 mL/min), and 6% with treated hypothyroidism [18••].

The commonest polypharmacy reported in the systematic review of TIH includes angiotensin II receptor blockers (ARBs) (59%, 95% CI 0 to 96%), angiotensin-converting enzyme (ACE) inhibitors (57%, 95% CI 29 to 83%), non-thiazide diuretics (e.g., loop- and potassium-sparing diuretics) (58%, 95% CI 19 to 91%), non-steroidal anti-inflammatory drugs (NSAIDs) (33%, 95% CI 18 to 49%), and anti-depressants (32%, 95% CI 19 to 47%). Whilst selective serotonin reuptake inhibitors (SSRIs) are also known to be associated with increased risk of developing hyponatremia, there was insufficient data to determine what proportion of the anti-depressant medication reported in TIH cases were SSRIs [17••]. The 2017 case series of hospitalized TIH patients broadly agreed with this polypharmacy data, with the most commonly co-prescribed medications being ACEi or ARB, non-thiazide diuretics, SSRIs (in 10%), aspirin, and NSAIDS [18••]. It is of course not surprising that other antihypertensive agents are commonly co-prescribed with thiazides, and both SSRIs and NSAIDs are also frequently used in this age group of patients, and hence whether or not co-prescription of these classes of groups increases TIH risk in those prescribed thiazides remains unclear.

## Time to Onset of TIH

**Fig. 1:**
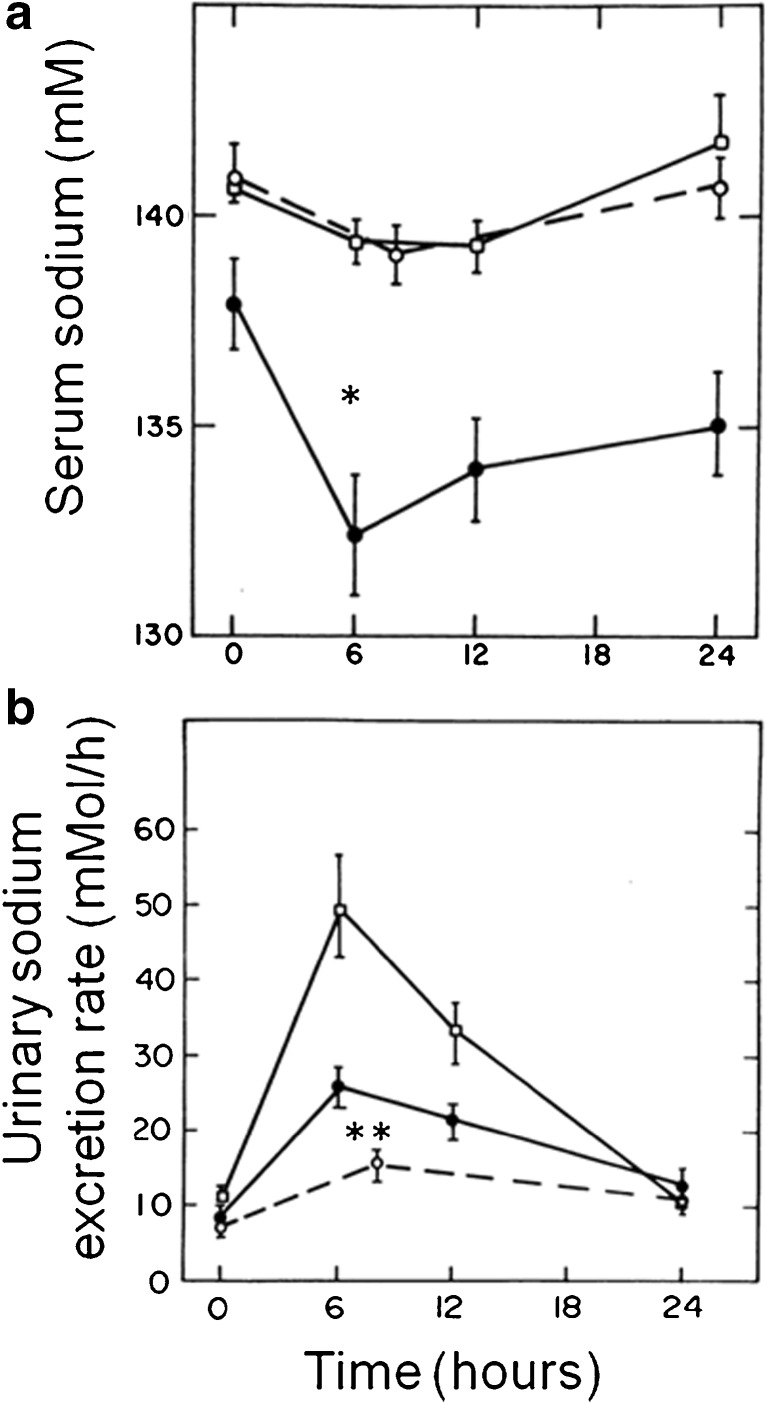
TIH can occur rapidly and is accompanied by excessive saliuresis [20•]. Rechallenge of 11 patients with previous TIH (closed circles), young controls (open squares), and age-matched elderly controls (open circles) with a single oral dose of hydrochlorothiazide 50 mg plus amiloride 5 mg. Serum sodium fell within 6 h in TIH patients only (**a**) (*P < 0.01) and this was accompanied by excessive saliuresis relative to age matched controls (**b**) (**P < 0.01). Reproduced with permission from Eitan Friedman, MD, et al., Thiazide-Induced Hyponatremia: Reproducibility by Single Dose Rechallenge and an Analysis of Pathogenesis. *Annals of Internal Medicine*, Jan 01, 1989 110

The interval between first thiazide ingestion and onset of hyponatremia in those developing TIH can be very variable. A study of single dose thiazide rechallenge in patients who had previously developed TIH showed that a reduction in serum sodium and increase in urinary sodium excretion were observed within hours (Fig. 1) [20•]. However, systematic review of the time to TIH found that clinical presentation with TIH occurred a mean of 19 (95% CI 8–30) days after starting treatment [17••].

This makes it difficult to determine what the optimal timing of a single post-thiazide initiation blood test for serum urea and electrolytes (U&E) should be. There is little consistency in the timing of this safety blood test but it is usually undertaken within a week or two of thiazide initiation. Whether undertaking more than one U&E blood test, e.g. at a few days and at 2–4 weeks, would improve detection of TIH before severe symptoms necessitate hospitalization is unclear. It is important however that all patients started on a thiazide receive at least one serum electrolyte check within a few weeks and it would seem prudent to undertake additional blood tests if symptoms of TIH occur before or after the routine blood test.

## Routine Blood and Urine Tests in TIH Clinical Care

It is usual to undertake blood and spot urine tests for U&E and other potential causes of hyponatremia when assessing a patient with suspected TIH. Our systematic review found that average mean trough serum U&E results in patients with TIH were sodium 117 mM (95% CI 114 to 120), potassium 3.3 mM (95% CI 3.0 to 3.5), and reduced osmolality of 242 mOsm/kg (95% CI 238 to 246 mOsm/kg) [17••]. The 2017 case series of hospitalized TIH patients broadly agreed with this with serum sodium 122 ± 0.6 mM, potassium 3.7 ± 0.06 mM and reduced osmolality of 255 ± 4 mOsm/kg [18••]. Plasma glucose was slightly higher in TIH patients than in controls but at 7.6 ± 0.4 mM is insufficiently high to cause pseudohyponatremia or to be the primary cause of hyponatremia.

Spot urinary U&E in the systematic review demonstrated less than maximal urinary dilution with urinary sodium concentration at 62 mM (95% CI 45 to 80 mM) and mean urine osmolality greater than plasma at 400 mOsm/kg (95% CI 366 to 434 mOsm/kg) [17••]. The 2017 case series also showed less than minimal spot urine concentration of 31 mM and inappropriately concentrated urine with osmolality greater than plasma at 366 mOsm/Kg [18••].

The phenotype of TIH is therefore similar to the criteria for SIADH outlined by Bartter and Schwartz including (1) decreased plasma osmolality (< 275 mOsm/kg), (2) inappropriately concentrated urine (> 100 mOsm/kg), (3) euvolemia on clinical examination, and (4) elevated urine Na (> 20 mEq/L) with (5) normal thyroid and adrenal function [21]. However, as discussed below, antidiuretic hormone (ADH) is normal or low in patients with TIH.

## Further Blood and Urine Phenotyping in TIH

In addition to the routine blood and spot urine tests undertaken in routine clinical care, TIH patients in the 2017 case series also underwent additional blood testing and 24 h urine collections both at the time of acute hyponatremia and after recovery to normonatremia following thiazide cessation [18••]. Hyponatremic TIH patients were also hypochloridemic, which is perhaps unsurprising since the thiazide-sensitive NCC transports both sodium and chloride. TIH cases also had a lower serum concentration of magnesium, calcium, zinc, and vitamin D relative to normonatremic controls. It is possible that the lower concentration of the non-sodium chloride electrolytes could represent a dilution of blood from water retention, and this was supported by increased fractional urate clearance, a marker of arterial blood volume expansion [22, 23]. The lower vitamin D levels in TIH cases might reflect the frailty of TIH patients who may be more limited in their diet and exposure to sunlight. Fortunately, all of these parameters improved substantially after thiazide cessation, although convalescent TIH cases showed persistence of a milder degree of hypochloridemia and hypozincemia relative to controls [18••].

TIH cases demonstrated an exaggerated free water reabsorption when taking thiazides compared to controls, which also supports a volume-expanded, diluted state. The continued production of urine which is more concentrated than plasma and which contains more than minimal sodium salt is physiologically inappropriate in the context of acute hyponatremia. Whilst 24 h urinary excretion of sodium and chloride was lower in acute TIH cases than in controls and increased from the acute state when convalescent, it is difficult to know whether such data indicates reduced salt intake in acute TIH cases given the dynamic pathophysiological state they are in. Convalescent TIH cases had slightly lower 24 h sodium and chloride excretion than controls but this was not statistically significant. There remains the possibility that TIH cases could have a slightly lower intake of dietary salt than controls but this remains unclear.

Given the similarity in phenotype between SIADH and TIH, antidiuretic hormone (ADH) was measured and was surprisingly lower in acute TIH cases than in controls or convalescent cases. The phenotype of TIH cases suggested ADH excess but in fact they had less ADH than controls, raising the question whether something other than ADH may be stimulating distal nephron water absorption.

## Genetic Predisposition to TIH

Thiazide therapy does not meaningfully alter average serum sodium concentration within the treated population with essential hypertension, suggesting that there are defined subgroups of patients who are susceptible to substantial reduction in sodium on exposure to thiazides. Genetic predisposition to TIH is supported by a high degree of reproducibility on single dose thiazide rechallenge where environmental factors such as sodium intake were controlled [20•]. A priori, the molecular mechanisms underlying predisposition to TIH must result in either diminished sodium reabsorption, inappropriate water retention or a combination of the two. That there may be non-NCC effects of thiazides contributing to TIH may be argued from the absence of significant hyponatremia in Gitelman syndrome [8] or Gitelman-mimic animals carrying a loss of function mutation in the NCC regulator Ste20 Proline-Alanine-rich Kinase (SPAK) [24].

A pilot genome-wide association study (GWAS) was undertaken in a discovery cohort of 48 patients hospitalized with severe TIH and compared to patients from the 1958 British birth cohort [18••]. Given the limited number of cases available a pre-defined cut off for signals of interest showing suggestive association of *P* < 10^−5^ was used. In total, 17 SNPs within 14 regions were identified as showing association with TIH at this level. The prostaglandin E2 (PGE2) transporter (PGT, coded by the *SLCO2A1* gene), was chosen for further study because TIH has a phenotype resembling SIADH, and it was the candidate with the best known function in regulating water reabsorption in the collecting duct via the AQP2 pathway. Sequencing confirmed the presence of a non-synonymous variant encoding p.A396T (rs34550074), which was in complete linkage disequilibrium with the sentinel GWAS SNP rs4854769, and the genetic association was replicated using a second cohort of TIH cases hospitalized with severe TIH [18••].

Immunohistochemistry of human cadaveric kidneys confirmed that PGT was expressed in the collecting duct and co-localized with AQP2. Using an in vitro cell expression system, PGT containing the phospho-mimic p.A396E demonstrated loss of function. This would be expected to reduce reuptake of PGE2 from the urinary lumen and indeed increased urinary PGE2 was seen in TIH patients with the PGT p.A396T variant compared to TIH patients who were wild type, suggesting that the PGT variant may have a functional effect in patients. Differences in urinary PGE2 resolved following thiazide cessation.

**Fig. 2:**
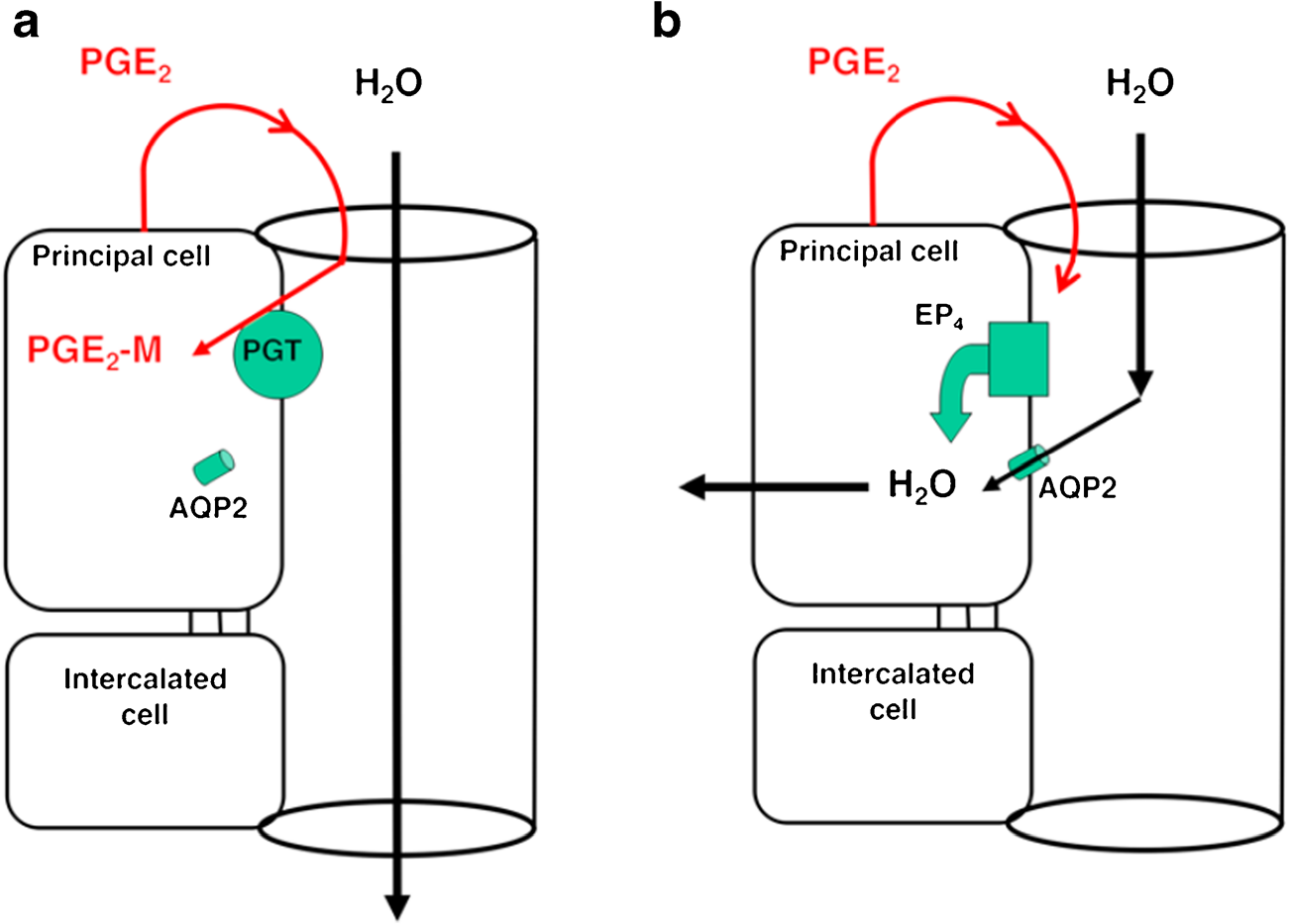
Hypothesis for the role of SLCO2A1 (also known as prostaglandin transporter, PGT) in contributing to thiazide-induced hyponatremia in individuals carrying the SLCO2A1 A396T variant. **a** Under low ADH conditions, apical PGT in the renal collecting duct scavenges PGE_2_ from the lumen, resulting in aquaporin-2 (AQP2) internalization and minimal osmotic water reabsorption. **b** With reduced or absent apical PGT, PGE_2_ reaching the lumen is able to stimulate apical EP4 receptors, resulting in insertion of AQP2 and osmotic water reabsorption [18••]

Taking these findings together, a hypothesis was proposed in which the combined effect of thiazide-induced impairment of renal free water generation, together with genetically determined PGE2-mediated increased water permeability of the collecting ducts, produces a combination of natriuresis and excessive water reabsorption sufficient to lead to a substantial decline in serum sodium concentration and presentation with severe TIH (Fig. 2).

## Management of TIH

The treatment of patients with TIH involves a careful balance between the desire to relieve symptoms by restoration of serum sodium and the risk of central pontine myelinolysis from overly rapid correction of serum sodium and other more general risks associated with unnecessary parenteral treatment. The balance of risks and benefits should be made by the treating physician with each patient’s circumstances considered individually. We would advocate using the cautious approach adopted for chronic hyponatremia with a maximum desirable rise in serum sodium of ≤ 10 mmol/l in the first 24 h and ≤ 8 mmol/l in each following 24 h. In frail, undernourished patients, it may be prudent to be even more cautious.

Our experience of managing TIH has been that cessation of the thiazide and avoidance of excessive water intake result in recovery of serum sodium at a clinically acceptable rate of a few millimoles per liter per day in most patients. Parenteral infusion of saline under closely monitored conditions is usually reserved for TIH cases with severe or life-threatening features. In support of this approach, an observational study of thiazide -associated hyponatremia found that progressively more aggressive strategies to elevate sodium in addition to thiazide cessation (including fluid restriction, parenteral infusion of normo- or hyper-tonic saline and the use of V_2_ antagonists such as tolvaptan) were found to be associated with increased probability of overly rapid correction of serum sodium [25]. It is important that patients with TIH have a serum electrolyte measurement after recovery to ensure that they are truly normonatremic. If chronic hyponatremia persists after thiazide withdrawal, then investigation for other causes of hyponatremia may be appropriate.

## Conclusion

Thiazide-induced hyponatremia is an important medical condition which is seen regularly in internal medicine and which causes substantial morbidity to often elderly frail patients. At present, the risk of developing TIH is largely unpredictable at the point of thiazide initiation. TIH is also an interesting and important condition scientifically as a relatively little studied paradigm of human sodium and water dysregulation which may provide an opportunity to learn more about the molecular processes underlying these essential aspects of renal physiology.

Pressing clinical issues include:

1. What is the optimal timing of a blood electrolyte test after starting thiazides, or should there be more than one electrolyte check?
2. Is it possible to identify a subgroup of patients at increased risk of TIH by developing a risk score based on their age, gender, weight, baseline blood, and urine tests, and genetic profile? If so, can this be used either to select alternative antihypertensive treatment or to focus additional monitoring if such patients are exposed to thiazides?
3. What is the optimal management of patients with TIH?

There are also many scientific questions remaining including:

1. What causes TIH in those who do not carry the PGT p.A396T mutation?
2. What role do the other genetic associations identified play in the pathophysiology of TIH?
3. What effect do drugs which alter the production of PGE2 or which antagonize the EP4 receptor have on TIH pathophysiology?
4. Is the cause of TIH consistent across ethnicities? Thiazides are used worldwide and yet most studies have been in Caucasians to date
5. What is the physiological response to non-thiazide diuretics in those with a history of TIH?

Given the effectiveness of thiazides in lowering blood pressure and preventing cardiovascular disease and their very low cost, it seems likely that thiazide-induced hyponatremia will be an important long-term issue in medicine; there are a potentially large number of patients who may benefit from greater understanding of this condition.
